# Gender differences in the prevalence, correlated factors and comorbidity of depression in adolescents: a cross-sectional study in Shanghai, China

**DOI:** 10.3389/fpubh.2024.1436413

**Published:** 2024-12-06

**Authors:** Yi Wang, Xiyan Zhang, Yan Li, Hongyun Qin, Xin Li

**Affiliations:** ^1^Clinical Research Center for Mental Disorders, Shanghai Pudong New Area Mental Health Center, School of Medicine, Tongji University, Shanghai, China; ^2^Shanghai Mental Health Center, School of Medicine, Shanghai Jiao Tong University, Shanghai, China

**Keywords:** depression, prevalence, correlated factors, comorbidity, gender difference

## Abstract

**Background:**

Prior studies have reported gender differences in the prevalence, comorbidity rates and related factors of depression during adolescence. But the gender differences in depression vary across studies. Besides, the evidence from Chinese adolescent samples is limited. This study aimed to explore gender differences in the depression-related factors, the potential interactions of the associated factors and clinical features of comorbid depression and anxiety among Chinese adolescents.

**Methods:**

A cross-sectional study involving 3,510 participants aged 11 to 16 years from schools (private and public) in Shanghai was conducted with an anonymous paper-and-pencil questionnaire. Children’s Depression Inventory (CDI), Child Anxiety Related Emotional Disorders (SCARED), Strengths and Difficulties Questionnaire (SDQ) were used to evaluate depression, anxiety and behavioral and emotional difficulties, respectively. Logistic regression model was used to explore the possible correlated factors of adolescent depression and analyze the interactions of the risk factors.

**Results:**

Our results showed that there was no gender difference in the prevalence rates of depression (*χ*^2^ = 0.047, *p* = 0.83), but the prevalence of depression in females began to exceed that of males at the ages of 15 and 16. The factors associated with depression varied by gender. Private school was a risk factor for depression only in boys (adjusted OR = 1.842 [95% CI, 1.286–2.636]), whereas girls from nuclear families (adjusted OR = 1.631 [95% CI, 1.156–2.300]) and other family structures (adjusted OR = 2.682 [95% CI, 1.502–4.788]) were more likely to experience depression compared to their peers in extended family structures. Interaction analyses showed there was a negative interaction between abnormal peer problems and 7th grade among boys (adjusted OR = 0.288 [95% CI, 0.086–0.951]). Furthermore, comorbidity rates of depression and anxiety were higher in girls than that in boys (*χ*^2^ = 14.457, *p* < 0.001). And girls with comorbidity showed increased SDQ peer problems scores (*Z* = −3.151, *p* = 0.002).

**Conclusion:**

The findings indicate it is important to develop gender-specific interventions for adolescent depression. And for boys, evaluating peer relationships may be particularly crucial in lower grades. Moreover, recognizing the gender-specific clinical features of comorbid depression and anxiety is important for appropriate clinical diagnosis and targeted treatment.

## 1 Introduction

Depression among adolescents has been a growing concern in recent years in China. A meta-analysis has revealed that the prevalence of depressive symptoms was 18.4% before 2000 and 26.3% after 2016 (1), and the results suggest that depressive symptoms in Chinese adolescents have increased significantly over time. Another research has indicated that the prevalence of depressive symptoms among children and adolescents was significantly associated with the region of China: the detected rates were 17.8% in the eastern region, 23.7% in the central region, 22.7% in the western region, and 14.5% in the northeastern region (2). Another meta-analysis also reported that the prevalence of depressive symptoms among elementary school students was highest in western China (3). The epidemiology of emotional problems, including depressive symptoms, is substantially influenced by socioeconomic background (4). Therefore, epidemiological studies of depressive symptoms in different regions with different socioeconomic backgrounds are of questionable generalizability and need to be studied separately.

Extensive studies have reported gender difference in the prevalence rates of adolescent depression, with females showing a relative predominance of depression that is evident globally. However, recent studies have shown that gender difference in adolescent depression differ across age groups. Depressive symptoms increase in early adolescence for females and late adolescence for males (5, 6). A recent meta-analysis suggests a small gender difference in depression prevalence between ages 8 and 11, followed by a sharp increase that peaks around age 16 and then stabilizes by early adulthood (7). Gonadal hormone changes, social role changes, pubertal status, and their interactions in adolescent girls may explain changes in the magnitude of age-specific gender difference (8, 9). The time course of the emergence of the gender difference in adolescence also showed national variations. A longitudinal study from New Zealand found that the prevalence of depression in females exceeded that of males between the ages of 13 and 15 (10). Studies in the United States indicated that gender difference in depression emerged prior to puberty (5). The data from Canada and the UK consistently demonstrated that gender difference in depression appeared at age 14 years (11). The study conducted in China revealed that girls had a higher level of depressive symptoms compared to boys in grade 9 (around 14.5 years old) (12). These cross-regions variations may be largely related to cultural differences (7). The above studies suggest that gender difference in the prevalence of depression among adolescents could be influenced by factors such as age and region. Although several epidemiological studies on adolescent depression have been carried out in eastern China (i.e., the eastern, northeastern, central, and western regions of China, categorized according to the official economic zone divisions) (13), few have examined the gender differences in depression prevalence among adolescents and how the differences evolve with age. And further exploration in this area is necessary.

Gender difference in the factors correlated with adolescent depression have also been extensively studied and confirmed (14). For example, evidence showed that there were gender differences in the associations between adverse childhood experiences and adolescent mental health symptoms, including depressive symptoms (15). Moreover, Anne and her colleagues have reported that a negative mother–child relationship predicted depressive symptoms only in girls, whereas school stress was associated with more depressive symptoms only in boys (14). However, few researchers have systematically explored gender-specific correlated factors of depression in Chinese adolescents. Besides, as we know, no previous study has explored the potential interactions of risk factors for adolescent depression.

As previously stated, there is a high rate of comorbid depression and anxiety among children and adolescents, with comorbidity estimates ranging from 15 to 75% (16). Evidence has shown that the comorbidity of depression and anxiety can lead to increased symptom severity and health costs and increases the risk of suicide (17, 18). The impairment associated with this comorbidity is often more pronounced than that associated with either symptom alone (19, 20). And researchers have found that girls have greater rate of comorbid depression and anxiety than boys (21). However, gender difference in the clinical characteristics of comorbid depression and anxiety and depression alone remain understudied. Information in this regard could be of importance to intervention efforts.

Notably, depression can have a significant impact on the overall well-being of adolescents. Depressed children are more likely to experience academic difficulties and have poorer outcomes later in life (22). Early recognition, intervention, and support are crucial in treating depression in adolescents. Since studies have consistently suggested that gender differences in the prevalence of depression, correlated factors and comorbidity among adolescents exist, we aimed to intensively examine the role of gender in (1) the factors correlated with depression, (2) the potential interactions of risk factors and (3) the clinical characteristics of comorbid depression and anxiety and depression alone in Chinese adolescents. And previous evidence has shown that family, school and interpersonal factors are usually associated with mental health in adolescents (23–25). Hence, we included factors of the three aspects and systematically explored gender-specific factors on adolescent depression. We examined the issues using a sample of adolescents from Shanghai Pudong New Area. As an emerging development zone, Pudong New Area, like other regions of eastern China, is characterized by rapid urbanization, rapid socioeconomic development, high income levels, and high population density (13). Thus, to some extent, the data on adolescent depression in Shanghai Pudong New Area can provide valuable insights into adolescent depression across the regions of eastern China.

## 2 Materials and methods

### 2.1 Participants and procedure

This was a cross-sectional study conducted between September and November 2021 in junior middle schools in Pudong New Area, Shanghai, China, using stratified random cluster sampling. First, all junior middle schools in Pudong New Area were stratified by school type (private school and public school), and then schools were drawn from the sampling frame of each type of school using the random number table method. The study enrolled 11 schools in total, including 2 private schools and 9 public schools. Second, all grades in the selected schools participated in the survey and two to three classes were randomly selected for each grade. After securing the cooperation of school administrators and obtaining informed consent from both parents and students, the anonymous paper-and-pencil questionnaire survey was conducted by trained researchers in in classroom settings. At the beginning of the survey, participants were assured that the data collected would be kept strictly confidential. The exclusion criteria were as follows: (1) refusal to participate in the study or failure to complete the informed consent form by the middle school students or their guardians; and (2) reports from parents or teachers indicating that the students had significant physical or mental health conditions.

### 2.2 Measures

#### 2.2.1 Socio-demographic variables

A self-designed demographic questionnaire was used to collect the participants’ general information, including age, gender, school type (public/private), grade, monthly family income (high/medium/low), family structure (nuclear family/extended family/other family structure, e.g., single-parent/restructured families), marital relationship of parents (harmony/disharmony), and parenting style (consistent/inconsistent). Self-reported peer relationship was assessed by means of the peer problems subscale of the Strengths and Difficulties Questionnaire (SDQ). The cutoff scores recommended for identifying peer problems in a child were as follows: normal: scores from 0 to 4; borderline: a score of 5; and abnormal: scores from 6 to 10 (26). Family economic status was categorized into three classes according to the *per capita* monthly family income: high (more than ¥10,000), medium (¥5,000–10,000), and low (less than ¥5,000).

#### 2.2.2 Depressive symptoms

Depressive symptoms were assessed using the Children’s Depression Inventory (CDI), which consists of 27 items with a total score ranging from 0 to 54. The CDI is a comprehensive multilayer assessment of depressive symptoms in children and adolescents that includes five subscales: negative mood, interpersonal problems, ineffectiveness, anhedonia and negative self-esteem. In this study, participants with a cutoff score of 19 or higher were considered to be depressed. The reliability and validity of the Chinese version of the CDI have been demonstrated in Chinese children and adolescents (27). The Cronbach’s alpha coefficient of the CDI was good (0.88) in the present study.

#### 2.2.3 Anxiety symptoms

Anxiety symptoms were assessed using the Screen for Child Anxiety Related Emotional Disorders, Child Version (SCARED-C), which consists of 41 items. Participants are asked to determine the frequency of each symptom during the last 3 months on a 3-point scale: 0 (almost never), 1 (sometimes), and 2 (often). A total score of 25 or higher is considered to indicate significant clinical anxiety (28). The scale is a reliable, valid, and sensitive measure for screening for anxiety disorders among children and adolescents (29). The Cronbach’s alpha coefficient of this questionnaire was 0.84 in the present study.

#### 2.2.4 Behavioral and emotional difficulties

Emotional and behavioral problems were assessed using the Chinese version of the self-report Strengths and Difficulties Questionnaire (SDQ-C). The questionnaire has 25 items and contains five subscales: emotional symptoms, conduct problems, hyperactivity-inattention, peer problems and prosocial behavior. Respondents score the items according to the severity of each statement (scores from 0 to 2: “not true” to “certainly true”). Except for prosocial behavior, the other four subscale scores are added to generate a total difficulties score. Higher scores on the scale indicate more serious emotional and behavioral problems (except for prosocial behavior). Previous evidence has indicated good reliability and validity for application of the scale in Chinese adolescents (30). The Cronbach’s alpha coefficient of the SDQ-C was 0.80 in this study.

### 2.3 Statistical analysis

Descriptive statistical analysis, *χ*^2^ tests, Mann–Whitney *U* tests and binary logistic regression were conducted using SPSS 26.0 (SPSS, Inc., Chicago, IL, United States). *χ*^2^ tests were conducted to examine gender differences in sociodemographic variables and confirm the effect of the seven factors including grade, school type, family economic status, consistency of parenting style, family structure, marital relationship of parents and self-reported peer problems on the prevalence rates of depression between the genders. Mann–Whitney *U* tests were conducted to examine the severity of depression and emotional/behavioral problems associated with different comorbid conditions among boys and girls. The multivariable logistic regression model was conducted to explore the possible risk and protective factors for depression. Factors that had an effect on the prevalence rate of depression in the chi-square test analysis were included in the regression model. We adjusted family economic status, consistency of parenting style, marital relationship of parents and family structure for adjusted OR among boys, and adjusted family economic status, consistency of parenting style, marital relationship of parents and school type for adjusted OR among girls.

Multiplicative and additive interactions were analyzed between associated factors, and the logistic regression model was used for both the multiplicative and additive interactions. Multiplicative interaction was presented as the product term and was measured by the odds ratio. When the odds ratio (OR) *>* 1, a positive multiplicative interaction is present (31). Additive interaction was measured by the Relative Excess Risk due to Interaction (RERI), with its 95% confidence intervals calculated using the delta method. When RERI *>*0, a positive additive interaction is present (32, 33). Interaction analyses were performed using R (version 4.4.0) with the “interactionR” packages. A *p-*value <0.05 was considered to indicate statistical significance, and all the statistical tests were two-sided.

## 3 Results

### 3.1 Socio-demographic characteristics

A total of 3,510 students aged 11–16 years were invited to participate in the survey and 3,395 questionnaires were collected, with a response rate of 96.7%. After excluding invalid data, 3,169 participants were selected in the analysis. There were 1,616 boys (51.0%) and 1,553 girls (49.0%). Descriptive statistics for all categorical variables are shown in Table 1. No differences were found among the variables, including grade, school type, family economic status, consistency of parenting style, family structure, marital relationship of parents between the genders (all *p* > 0.05). However, self-reported peer problems (normal/borderline/abnormal) were significant different between boys and girls (*p* < 0.001).

**Table 1:** Socio-demographic characteristics of the sample (*N* = 3,169).

Variables	Adolescent boys (*N* = 1,616)	Adolescent girls (*N* = 1,553)	*χ*^2^/*Z*	*p*-value
**Age**	13(12,14)	13(12,14)	−1.228	0.219
**Grade**			2.915	0.405
6th grade	308(19.1%)	296(19.1%)		
7th grade	455(28.2%)	406(26.1%)		
8th grade	417(25.8%)	438(28.2%)		
9th grade	436(27%)	413(26.6%)		
**School type**			1.274	0.259
Public school	1,364(84.4%)	1,333(85.8%)		
Private school	252(15.6%)	220(14.2%)		
**Family economic status**			1.451	0.484
High	227(14%)	210(13.5%)		
Medium	831(51.4%)	775(49.9%)		
Low	558(34.5%)	568(36.6%)		
**Consistency of parenting style**			1.008	0.315
Consistent	1,133(70.1%)	1,114(71.7%)		
Inconsistent	483(29.9%)	439(28.3%)		
**Family structure**			0.483	0.785
Nuclear family	983(60.8%)	950(61.2%)		
Extended family	494(30.6%)	461(29.7%)		
Other family structures	139(8.6%)	142(9.1%)		
**Marital relationship of parents**			0.069	0.793
Harmony	1,427(88.3%)	1,376(88.6%)		
Disharmony	189(11.7%)	177(11.4%)		
**Self-reported peer problems**			37.616	<0.001
Normal	1,304(80.7%)	1,374(88.5%)		
Borderline	175(10.8%)	109(7.0%)		
Abnormal	137(8.5%)	70(4.5%)		

### 3.2 Prevalence of depression according to demographic factors in boys and girls

**Figure 1:**
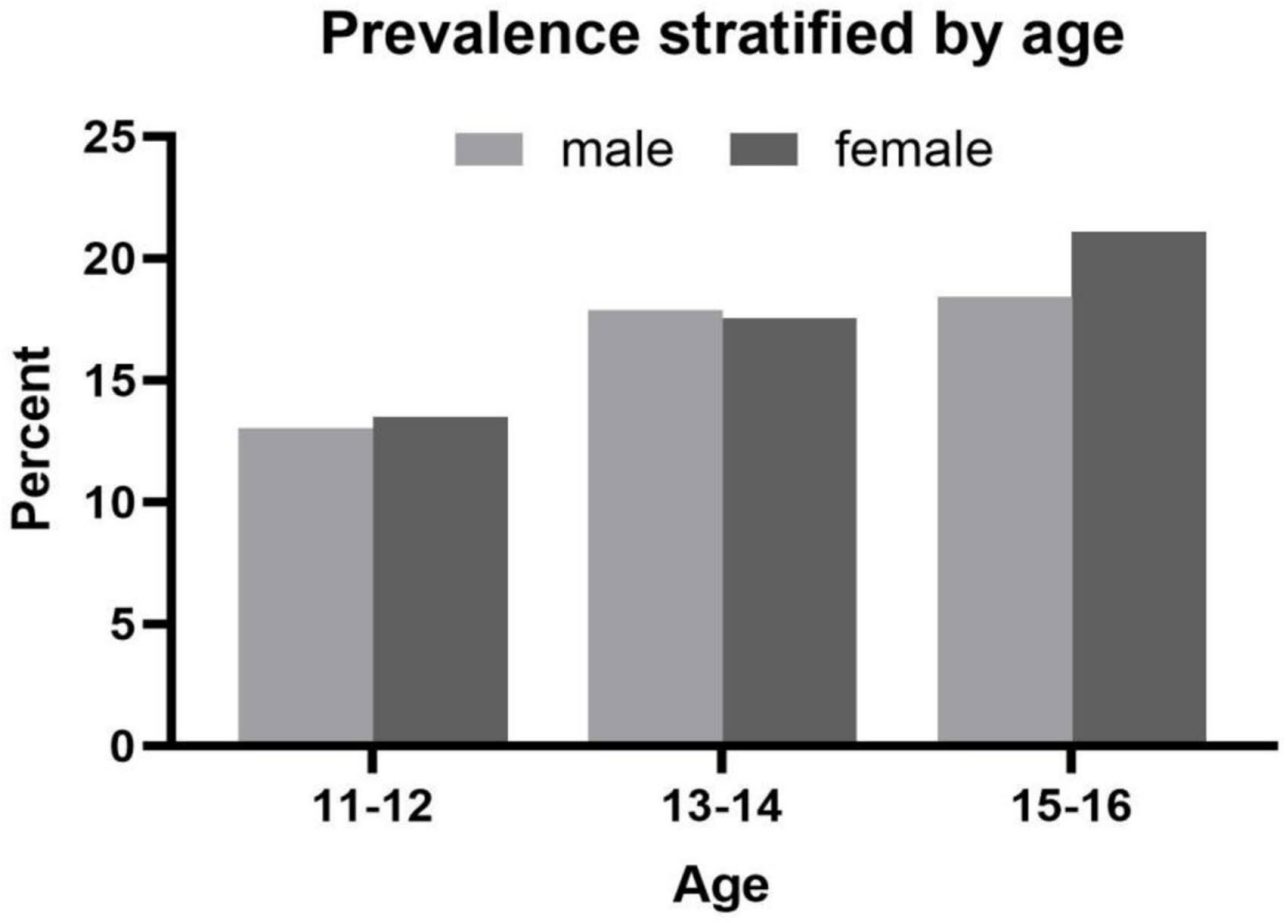
Prevalence stratified by age.

The prevalence rate of depression in adolescents was 16.72% (16.60% in boys and 16.90% in girls) and did not differ by gender (*χ*^2^ = 0.047, *p* = 0.83). Further statistics were conducted by age stratification. As shown in Figure 1, the prevalence tended to increase with age for both female and male adolescents. Although no significant gender difference in depression prevalence was found in different age subgroups, we observed that the prevalence of depression among females started to exceed that of males at the ages of 15–16 (21.6% vs. 18.9%).

For boys, the prevalence rates of depression significantly differed by grade (*χ*^2^ = 8.790, *p* = 0.032), school type (*χ*^2^ = 7.860, *p* = 0.005) and self-reported peer problems (*χ*^2^ = 167.702, *p <* 0.001). No significant differences were found among the following variables, including family economic status, consistency of parenting style, family structure or marital relationship of parents (all *p* > 0.05) (Table 2).

**Table 2:** Differences of prevalence of depression according to demographic factors.

Variable	Boys without depression (*N* = 1,348)	Boys with depression (*N* = 268)	*χ* ^2^	*p*-value	Girls without depression (*N* = 1,291)	Girls with depression (*N* = 262)	*χ* ^2^	*p*-value
**Grades**			8.790	0.032			19.770	<0.001
6th graders	274 (89.0%)	34 (11.0%)			267 (90.2%)	29 (9.8%)		
7th graders	372 (81.8%)	83 (18.2%)			334 (82.3%)	72 (17.7%)		
8th graders	346 (83.0%)	71 (17.0%)			369 (84.2%)	69 (15.8%)		
9th graders	356 (81.7%)	80 (18.3%)			321 (77.7%)	92 (22.3%)		
**School type**			7.860	0.005			0.314	0.575
Public school	1,153 (84.5%)	211 (15.5%)			1,111 (83.3%)	222 (16.7%)		
Private school	195 (77.4%)	57 (22.6%)			180 (81.8%)	40 (18.2%)		
**Family economic status**			0.855	0.652			1.327	0.515
High	194 (85.5%)	33 (14.5%)			176 (83.8%)	34 (16.2%)		
Medium	692 (83.3%)	139 (16.7%)			651 (84%)	124 (16%)		
Low	462 (82.8%)	96 (17.2%)			464 (81.7%)	104 (18.3%)		
**Consistency of parenting style**			0.077	0.781			0.212	0.645
Consistent	947 (83.6%)	186 (16.4%)			923 (82.9%)	191 (17.1%)		
Inconsistent	401 (83.0%)	82 (17%)			368 (83.8%)	71 (16.2%)		
**Family structure**			1.068	0.586			11.476	0.003
Extended family	416 (84.2%)	78 (15.8%)			406 (88.1%)	55 (11.9%)		
Nuclear family	813 (82.7%)	170 (17.3%)			771 (81.2%)	179 (18.8%)		
Other family structures	119 (85.6%)	20 (14.4%)			114 (80.3%)	28 (19.7%)		
**Marital relationship of parents**			1.237	0.266			0.678	0.410
Harmony	1,185 (83.0%)	242 (17.0%)			1,140 (82.8%)	236 (17.2%)		
Disharmony	163 (86.2%)	26 (13.8%)			151 (85.3%)	26 (14.7%)		
**Self-reported peer problems**			167.702	<0.001			149.412	<0.001
Normal	1,164 (89.3%)	140 (10.7%)			1,199 (87.3.%)	175 (12.7%)		
Borderline	106 (60.6%)	69 (39.4%)			61 (56%)	48 (44%)		
Abnormal	78 (56.9%)	59 (43.1%)			31 (44.3%)	39 (55.7%)		

For girls, the prevalence rates of depression significantly differed by grade (*χ*^2^ = 19.770, *p <* 0.001), family structure (*χ*^2^ = 11.476, *p* = 0.003) and self-reported peer problems (*χ*^2^ = 149.412, *p <* 0.001). No significant differences were found among the following variables, including school type, economic status, consistency of parenting style or marital relationship of parents (all *p* > 0.05) (Table 2).

### 3.3 Correlated factors of adolescent depression

Multivariable logistic regression analysis showed that attending a private school (public school as a reference, adjusted OR = 1.861 [95% CI, 1.298–2.668]), being in a senior class (6th grade as a reference, 7th grade: adjusted OR = 1.898 [95% CI, 1.208–2.983]; 8th grade: adjusted OR = 1.801 [95% CI, 1.133–2.863]; and 9th grade: adjusted OR = 2.075 [95% CI, 1.310–3.285]), and having poor peer problems (normal peer relationships as a reference, borderline: adjusted OR = 5.616 [95% CI, 3.930–8.025]; abnormal: adjusted OR = 6.412 [95% CI, 4.353–9.445]) were risk factors for depression in boys. For girls, being in a senior class (7th grade: adjusted OR = 2.215 [95% CI, 1.357–3.614]; 8th grade: adjusted OR = 1.892 [95% CI, 1.160–3.086]; and 9th grade: adjusted OR = 3.052 [95% CI, 1.888–4.932]) and having poor peer problems (borderline: adjusted OR = 5.623 [95% CI, 3.682–8.589]; abnormal: adjusted OR = 9.670 [95% CI, 5.755–16.248]) were risk factors for depression. Girls with nuclear (adjusted OR = 1.631 [95% CI, 1.156–2.300]) and other family structures (adjusted OR = 2.682 [95% CI, 1.502–4.788]) were more prone to depression compared to those in an extended family structure (Table 3).

**Table 3:** Possible risk and protective factors of depression for boys (*n* = 1,616) and girls (*n* = 1,553).

Variables	*B*	SE	Adjusted OR	95% CI	*p*-value
Boys					
**School type (public school as reference)**	0.621	0.184	1.861	1.297–2.668	0.001
**Grade (6th grade as reference)**					0.014
7th grade	0.641	0.231	1.898	1.208–2.983	0.005
8th grade	0.588	0.236	1.801	1.133–2.863	0.013
9th grade	0.730	0.234	2.075	1.310–3.285	0.002
**Self-reported peer problems (normal peer relationship as reference)**					<0.001
Borderline	1.726	0.182	5.616	3.930–8.025	<0.001
Abnormal	1.858	0.198	6.412	4.353–9.445	<0.001
Girls					
**Grade (6th grade as reference)**					<0.001
7th grade	0.795	0.250	2.215	1.357–3.614	0.001
8th grade	0.638	0.250	1.892	1.160–3.086	0.011
9th grade	1.116	0.245	3.052	1.888–4.932	<0.001
**Family structure (Extended family as reference)**					0.001
Nuclear family	0.489	0.175	1.631	1.156–2.300	0.005
Other family structures	0.987	0.296	2.682	1.502–4.788	0.001
**Self-reported peer problems (normal peer relationship as reference)**					<0.001
Borderline	1.727	0.216	5.623	3.682–8.589	<0.001
Abnormal	2.269	0.265	9.670	5.755–16.248	<0.001

Family economic status, consistency of parenting style, marital relationship of parents and family structure were adjusted for adjusted OR among boys.

Family economic status, consistency of parenting style, marital relationship of parents, school types were adjusted for adjusted OR among girls.

### 3.4 Interaction analysis of associated factors

Table 4 showed the results of multiplicative interaction analysis of grades and peer problems. A negative interaction was observed between abnormal peer problems and 7th grade among boys (adjusted OR = 0.288 [95% CI, 0.086–0.951]). No significant interactions were found for other risk factors either in boys or girls, regardless of whether using multiplicative or additive models.

**Table 4:** Interaction analysis of grade and peer problems.

Variables	*B*	SE	Adjusted OR	95% CI	*p*-value
Grade (6th grade as reference)					
7th grade	0.856	0.310	2.353	1.311–4.456	0.006
8th grade	0.598	0.325	1.818	0.978–3.529	0.066
9th grade	0.931	0.314	2.538	1.402–4.840	0.003
Self-reported peer problems (normal peer relationship as reference)					
Borderline	1.655	0.491	5.235	1.927–13.488	0.001
Abnormal	2.558	0.487	12.916	4.951–33.914	0.000
Grade × peer problems					
Borderline × 7th grade	0.084	0.592	1.088	0.344–3.56	0.887
Abnormal × 7th grade	−1.244	0.611	0.288	0.086–0.951	0.042
Borderline × 8th grade	0.377	0.601	1.457	0.454–4.849	0.530
Abnormal × 8th grade	−0.363	0.628	0.696	0.202–2.392	0.564
Borderline × 9th grade	−0.181	0.602	0.834	0.258–2.777	0.763
Abnormal × 9th grade	−0.833	0.608	0.435	0.131–1.433	0.171

School type, family economic status, consistency of parenting style, marital relationship of parents and family structure were adjusted for the regression model.

### 3.5 Comorbidity rates and emotional/behavioral problems among depressed adolescents with and without comorbid anxiety

A total of 67.36% of the potential depressed adolescents had comorbid anxiety, and 32.64% had depression alone. Among the boys, 59.70% of the potential depressed students had comorbid anxiety, and among the girls, 75.20% had comorbid anxiety. The prevalence rate of comorbid depression and anxiety in girls was higher than that in boys (*χ*^2^ = 14.457, *p <* 0.001).

**Figure 2:**
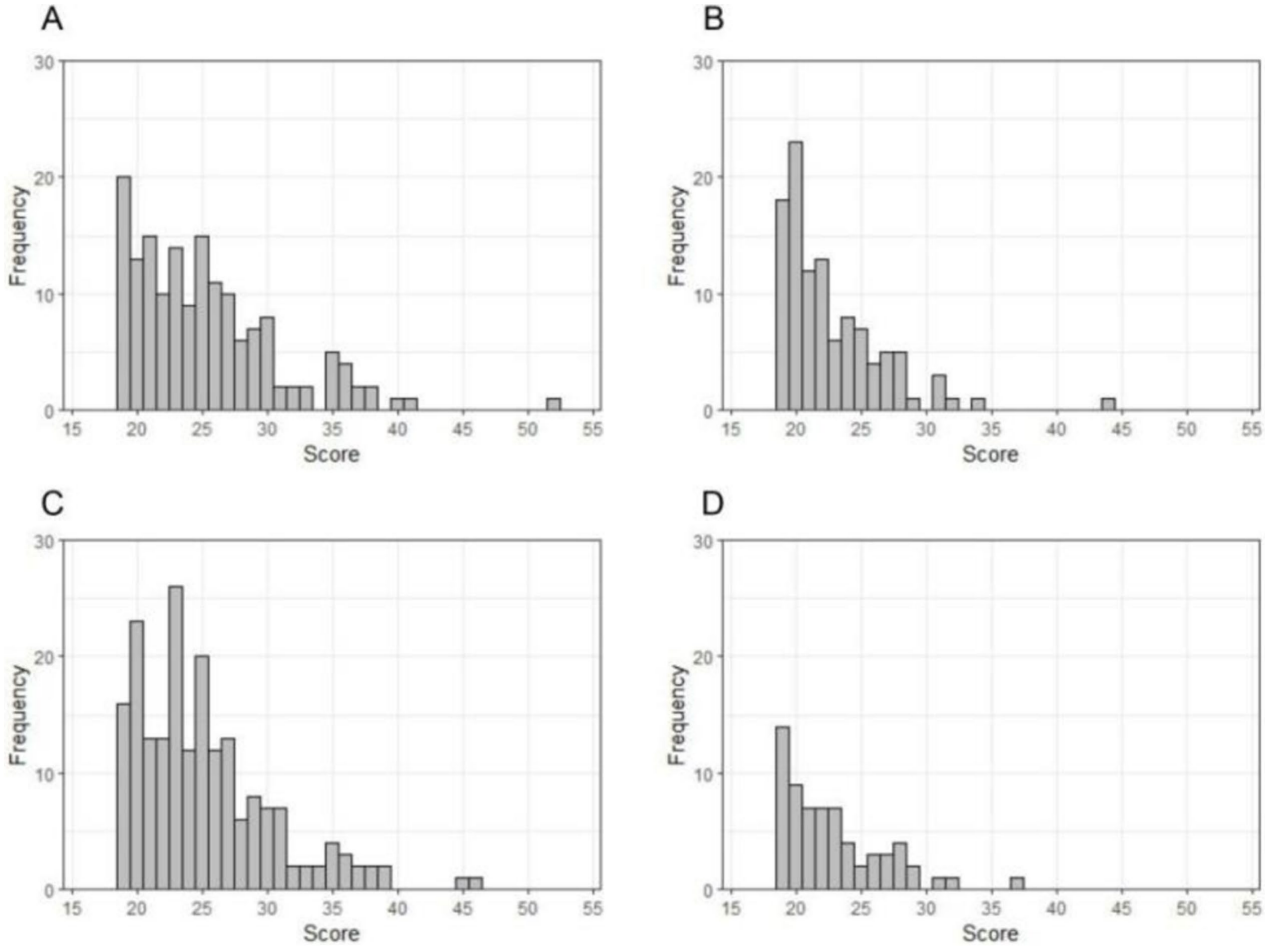
Children’s Depression Inventory (CDI) scores distributions among boys with comorbid conditions **(A)**, boys with depression alone **(B)**, girls with comorbid conditions **(C)** and girls with depression alone **(D)**.

Figure 2 showed CDI scores distributions among adolescents with comorbid depression and anxiety and depression alone. Both boys [24 (21, 28) vs. 22 (20, 25), *Z* = −4.035, *p <* 0.001] and girls [24 (21, 27.50) vs. 22 (20, 25), *Z* = −3.570, *p <* 0.001] who had comorbid depression and anxiety showed more severe depressive symptoms than those with depression alone.

Boys with comorbid depression and anxiety had higher scores on the SDQ emotional symptoms (*Z* = −7.640, *p* < 0.001), hyperactivity-inattention problems subscales (*Z* = −3.376, *p* = 0.001) and higher total difficulties scores (*Z* = −5.328, *p* < 0.001) than boys with depression alone. Girls with comorbid depression and anxiety had higher scores on the SDQ emotional symptoms (*Z* = −6.996, *p* < 0.001), hyperactivity-inattention problems (*Z* = −3.820, *p* < 0.001) and peer problems (*Z* = −3.151, *p* = 0.002) subscales and higher total difficulties scores (*Z* = −5.777, *p* < 0.001) than girls with depression alone (Table 5).

**Table 5:** Behavioral and emotional difficulties among depressed adolescents with and without comorbidity.

SDQ Scale (Median [M25, M75])	Boys with comorbid conditions (*N* = 160)	Boys with depression alone (*N* = 108)	*Z*	*p*-value	Girls with comorbid conditions (*N* = 197)	Girls with depression alone (*N* = 65)	*Z*	*p*-value
Emotional symptoms	6(4,7)	3(2,5)	−7.64	<0.001	6(4.5,7)	4(2,5)	−6.996	<0.001
Hyperactivity inattention problems	5(4,7)	5(3,6)	−3.376	0.001	5(4,7)	4(2.5,5)	−3.820	<0.001
Prosocial behavior	7(5,8)	7(5,9)	−0.818	0.413	7(6,9)	6(5,9)	−0.862	0.389
Conduct problems	3(2,5)	3(2,4)	−1.059	0.289	3(1,4)	2(2,4)	−0.658	0.511
Peer problems	4(3,5)	5(3,5)	−0.370	0.712	4(2.5,5)	3(2,4)	−3.151	0.002
Total difficulties scores	19(16,22)	15(12,18.75)	−5.328	<0.001	18(15,21)	13(10,17)	−5.777	<0.001

## 4 Discussion

Our study showed that the prevalence rate of depression in our sample was 16.72%, which was much lower than the prevalence rate of children and adolescents in Chongqing (western China), which also utilized the CDI scale to assess adolescent depression (34). The difference of prevalence rates may be explained by different economic conditions, living environments and social support system in different regions of China (2). Besides, our results indicated no significant difference in depression prevalence between boys and girls, which were consistent with a previous study conducted on Chinese adolescents aged 10–15 years (35). However, we found that at the ages of 15–16 years, female adolescents began to have a higher prevalence than male adolescents, which is consistent with the findings of the study conducted in Shenzhen (12). The two researches conducted in Shanghai and Shenzhen, both categorized as eastern cities in China, exhibited similar findings in terms of gender difference in depression rates. The similarities suggest that our results can, to some extent, be generalized to other regions within eastern China. However, the prevalence rate of depression among girls in Shanghai exceeded that of boys at a slightly later age than in Chongqing, which similarly found no gender difference in prevalence rates of depression among adolescents who were 7–17 years old, but noted a higher prevalence of depression among girls compared to boys between the ages of 14–17 years (34). The findings imply that there are variations in gender differences in the prevalence of depression between eastern and western regions, and further research on gender difference in different regions is needed. Existing evidence indicates there are gender-age and gender-region patterns in the development of depression and cultural differences and socio-economic status account for the variability in the emergence and development of gender differences in depression (7, 36). These studies indicate that we should take a developmental perspective in understanding gender difference in prevalence of depression and the gender differences vary across regions with different economic and cultural contexts.

Our multivariable logistic regression analysis showed that the factors correlated with depression were different between boys and girls. Adolescent boys in private schools were more likely to be depressed. This might be due to more academic pressure in private schools in Shanghai, and the results suggested that boys were more vulnerable to school pressures compared to girls, which was consistent with the results of Anne Kaman’s research (14). Furthermore, we found that girls from nuclear or other family structures were more prone to depression compared to those from extended families. And evidence has also shown that living together with grandparents is beneficial for children’s and adolescents’ health (37). Due to the competitive environment, parents show strong expectations for academic achievement of their children (34), whereas grandparents might be more concerned about the children’s mood, diet and daily life. In extended families, adolescents typically have a more diverse set of relationships and this can create a more laid-back and relaxing family atmosphere. And compared to boys, girls are more susceptible to family trauma (38, 39). Hence, girls might be more sensitive to family emotional climate which can be affected by family structures.

Being in a senior class was a risk factor for depression in both boys and girls. The possible explanation may be that as student progress through higher grades, academic pressure increases. And academic pressure is associated with adolescent mental health problems (40). Besides, peer relationships played important roles in adolescent depression in both boys and girls. Poor peer relationships can lead to difficulties in regulating emotions (41) and disrupt development of psychological adjustment behaviors (42). These disruptions in normal emotional and behavioral regulation are highly likely to lead to depression. Thus, developing social skills training programs for adolescents may have a positive impact on their emotional regulation.

However, we did not find that depressive symptoms of adolescents were associated with family income, despite several studies indicating a link between family economic status and adolescent depression (25, 43). The discrepancies of the results may be due to the insufficient assessment of the family economic status of our sample. Given that Shanghai, as China’s leading global city, attracts numerous migrants from other cities seeking employment opportunities (44), and with housing prices ranking among the highest in the nation (45), many migrants are burdened with huge expenses for purchasing or renting a house, resulting in carrying a high mortgage. Therefore, relying solely on family income may not provide a comprehensive assessment of the family economic conditions. Furthermore, migration is a variable that may not only be associated with the evaluation of a family’s economic situation, but also may be linked to the adolescent’s social support network and acculturation process. Hence, the correlation of migration and adolescent depression should be further explored in the future. Additionally, it is possible that disparities in research findings regarding the correlation between family income and adolescent depression may be attributed to factors such as psychological resilience. Evidence has indicated that the relationship between risk factors and depression can be moderated by resilience (46, 47). And further research on psychological resilience of adolescents and its role in depression is needed.

There was a negative multiplicative interaction between 7th grade and abnormal relationships among boys, indicating a reduced risk of depression associated with abnormal relationships in 7th grade compared to 6th grade. Due to lower academic stress in 6th grade compared to 7th grade, male adolescents may concentrate more on academic achievement as they progress to senior grades (48), resulting in peer problems having a diminished impact on their depressive symptoms. The results showed that the odds ratios (ORs) for the interactions between abnormal relationships and higher grades (including 8th and 9th grades) are all below 1. Although the results did not reach statistical significance in the 8th or 9th grades, it can be hypothesized that abnormal problems have less impact on depressive symptoms in male adolescents in the senior grades compared to those in the 6th grade. Nevertheless, for adolescent girls, maintaining relationships has always been a crucial part of their lives (49). They may prioritize their peer connections despite the academic pressures associated with entering into higher grades. The findings suggest that for adolescent boys, focusing on adolescent peer relationships may be particularly crucial in lower grades. And tailoring intervention strategies to the specific needs of different grades could enhance the effectiveness of addressing adolescent depression.

Analyses of comorbidity showed that the prevalence of comorbidity in girls was greater than that in boys, which was consistent with previous research. A study of a sample of 12- to 17-year-old American adolescents showed that the prevalence of comorbid anxiety and depression in girls (28.4%) was significantly greater than that in boys (17.1%) (21). The possible explanation may be that girls are more likely to present with internalizing problems (e.g., depressive and anxiety symptoms) than boys (50).

Moreover, our findings indicated that more depressive symptoms and behavioral problems occurred in the comorbid group, which was consistent with previous findings. Studies have confirmed that comorbid anxiety and depression is associated with greater impairment and symptom severity related to the primary diagnosis (17, 18). Adolescents with comorbid depression and anxiety had more hyperactivity-inattention problems, possibly because these adolescents had more depressive symptoms, and difficulty in concentrating is a common clinical symptom of depression. The result that girls with comorbid conditions had more peer problems might also be due to these girls experiencing more severe depressive symptoms. Researchers have suggested that individuals with depression are more likely to experience increased peer relationship stress, and depression can affect a person’s ability to maintain healthy relationships (51). While a longitudinal study has shown that when adolescents experience interpersonal stress in relationships with peers, subsequent depression may occur (52). Therefore, we assume that there may be a two-way relationship between depression and peer relationships and this speculation needs to be examined in a longitudinal study.

## 5 Limitations

The findings should be interpreted with caution because of some limitations. First, the sample of this study was from urban areas of Shanghai, thus the findings may only be generalized to urban areas of eastern China and not to other regions outside of eastern China. Second, it was a cross-sectional design. The results of the present study can provide insights into associations between variables, but the temporal order of events cannot be demonstrated. Third, the self-designed demographic questionnaire items were solely based on the subjective feelings of the adolescents’ parents, which might not reflect objective reality. Fourth, we did not account for the impact of the migration phenomenon on adolescent depression in Shanghai, nor did we consider factors such as psychological resilience, potentially limiting the comprehensiveness of our exploration of adolescent depression in the city. Therefore, stronger designs and more covariates should be considered to identify the factors correlated with adolescents’ depression.

## 6 Conclusion

In conclusion, our results showed that the prevalence rates of depression were similar between the boys and girls, whereas girls started to experience higher rates of depression compared to boys at the ages of 15–16. The factors correlated with depression were not the same between the genders. And peer problems are associated with a lower risk of depression in senior boys compared to boys in 6th grade. For depressed boys who present with more hyperactivity-inattention problems and depressed girls who have more hyperactivity-inattention problems and interpersonal issues, attention should be paid to whether they co-occur with anxiety. Addressing this comorbidity in depressed adolescents is crucial for appropriate diagnosis and targeted treatment. Our research provides a basis for formulating gender-specific intervention policies for junior high school students and provides insight into gender-specific clinical features of comorbidity for adolescents in urban areas of eastern China.

## Data Availability

The original contributions presented in the study are included in the article/supplementary material, further inquiries can be directed to the corresponding author.
